# First study on assessments of farmers’ benefits under a payment program based on dairy milk quality in Thailand

**DOI:** 10.14202/vetworld.2022.1051-1057

**Published:** 2022-04-24

**Authors:** Veerasak Punyapornwithaya, Katechan Jampachaisri, Orapun Arjkumpa, Methanon Moonpho, Kunnanut Klaharn, Naovarat Kampoosiri, Chalutwan Sansamur

**Affiliations:** 1 Veterinary Public Health and Food Safety Centre for Asia Pacific, Faculty of Veterinary Medicine, Chiang Mai University, Chiang Mai 50100, Thailand; 2 Research Group for Veterinary Public Health, Faculty of Veterinary Medicine Chiang Mai University, Chiang Mai 50100, Thailand; 3 Department of Mathematics, Faculty of Science, Naresuan University, Phitsanulok 65000, Thailand; 4 Department of Livestock Development, Animal Health Section, The 4^th^ Regional Livestock Office, Khon Kaen, Thailand; 5 Veterinary Research and Development Center, Upper Northern Region, Lampang 52190, Thailand; 6 Bureau of Livestock Standards and Certification, Department of Livestock Development, Bangkok 10400; Thailand; 7 Bureau of Quality Control of Livestock Products, Department of Livestock Development, Mueang Pathum Thani District, Pathumthani 12000, Thailand; 8 Akkhararatchakumari Veterinary College, Walailak University, Nakhon Si Thammarat, 80160, Thailand

**Keywords:** benefits, bulk tank milk, milk compositions, milk quality, payment program, somatic cell counts

## Abstract

**Background and Aim:**

To improve overall milk quality in Thailand, dairy farmers and milk collection centers employ a payment program based on milk quality (PPBMQ) for milk trade. This study aimed to determine and compare the proportion of dairy farmers receiving benefits from the PPBMQ using data from selected dairy cooperatives located in northern and central regions in Thailand.

**Materials and Methods:**

Monthly data on milk components (n=37,077), including fat, solids not fat (SNF), and somatic cell counts (SCC) were collected from the two regions in 2018 and 2019. Based on the PPBMQ, farmers were classified into benefit-gain, benefit-loss, and no-benefit groups. A mixed-effects logistic regression model was used to compare the number of farmers in northern and central regions who received monthly benefits from the PPBMQ.

**Results:**

More than 70% of dairy farmers benefited from the PPBMQ. The proportion of dairy farmers in the benefit-gain group was higher in the northern region (88.7%) than in the central region (57.1%). A high percentage of dairy farmers in the central region lost their benefits mainly due to SCC (40%) and SNF (44%).

**Conclusion:**

The PPBMQ benefited the vast majority of dairy producers in the northern region and approximately two-thirds of those in the central region. Thus, the efforts of authorities and stakeholders should be enhanced to support dairy farmers in the central region in improving milk quality.

## Introduction

In Thailand, the dairy product industry is a promising sector as it generates income for farmers; in 2018, 1.29 million tons of raw milk was produced, with the average milk price per kilogram (a unit used in Thailand) was 18.30 baht (0.6 USD) or 59.55 USD per 100 kg [1]. The principal regions of dairy farming include the central, northeast, and northern. In 2020, there were registered 806,441 heads of dairy cattle raising by 24,252 dairy farms [2]. Most dairy farms are operated by smallholder farmers. Crossbreeding over 75% Holstein with 25% or less local cattle has been widely used across Thailand [3,4]. Dairy cooperatives were created with support from government authorities to regulate milk trading between farmers and factory milk purchasers. The dairy cooperative operates a milk collection center in which bulk tank milk (BTM) is obtained from the dairy farmers. The cooperative buys milk from the farmers and then sells it to milk processing companies [3,5]. In 2019, 161 milk collection centers were located in different regions of the country: Approximately 58% belonged to dairy cooperatives and the rest were operated by private companies [1].

Thailand aimed to enhance the quality of milk produced in the country [6]. The government established a payment program based on milk quality (PPBMQ) in 2016 to encourage dairy farmers to produce high-quality milk, and it is now widely utilized across the country [7]. According to the PPBMQ, dairy products have three classes based on milk composition, including fat, solids not fat (SNF), and somatic cell counts (SCC), and each class has a corresponding rate based on their value. The payment programs can be further classified into three categories: base, bonus, and penalty. As fat and SNF are essential nutrients [8], the higher the amount of fat and SNF in milk, the higher the price. In contrast, since SCC negatively affects milk shelf life [9] and milk products [10-12], a penalty is imposed on milk with high SCC levels. The PPBMQ is used in several countries for milk commerce between buyers and dairy farmers [13-17]. Some studies on PPBMQ determined the association between the implementation of PPBMQ and the improvement of milk quality [14,18], while others reported the relationship between farm economics and payment programs [15]. As the PPBMQ could result in gains or losses for Thai dairy farmers, it is critical to ascertain the number of farmers who could benefit or lose from the payment scheme.

Thus, this study aimed to determine and compare proportions of dairy gaining benefits from the PPBMQ using data from two important dairy production regions of Thailand.

## Materials and Methods

### Ethical approval

Ethical approval for animal research was not required because live animals were not involved in this study. Milk sample collections and laboratory works were performed by authorized personnel from government sectors under the policy of the Department of Livestock Development (DLD), Thailand and thus additional permission was not required. The authors played no role in milk sampling and laboratory analysis. The authors only used milk quality data, thereby preserving the farmers’ privacy.

### Study period and area

**Figure-1:**
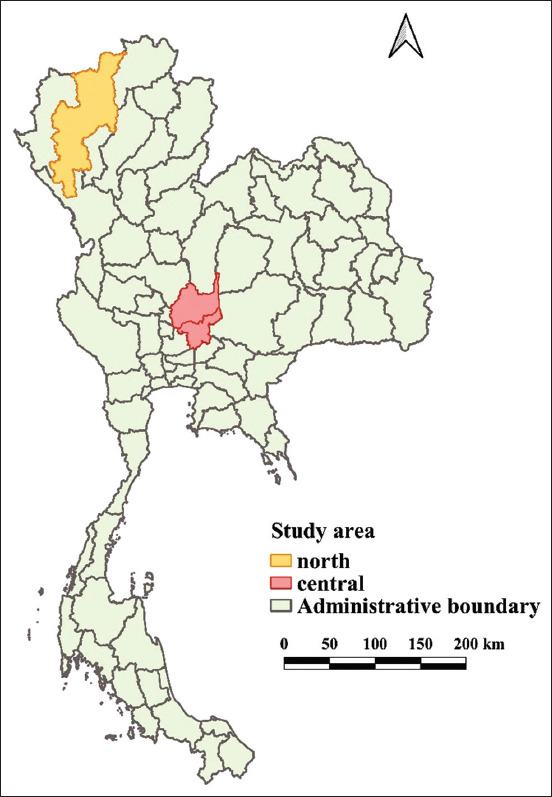
A map depicting the study provinces in the northern and central regions of Thailand. Milk samples were collected from dairy farms located in these provinces. The map was created using QGIS (version 2.18.28), QGIS Geographic Information System, Open Source Geospatial Foundation Project. All content is licensed under Creative Commons Attribution ShareAlike 3.0 license (CC BY-SA), available at http://www.qgis.osgeo.org.

The study was conducted from January 2018 to December 2019. Dairy cooperatives located in the northern (Chiang Mai province) and the central (Saraburi and Lopburi provinces) regions participated in this study (Figure-1). These cooperatives meet the criteria of being among the top five milk producers in the province, having been in business for more than two decades, and participating in the DLD milk quality testing program. According to Thai qualities and standards of raw cow milk, raw cow milk is used as raw material in the production of cow milk, flavored milk, and other milk products to protect the consumer and maintain food safety. SCC shall not exceed 500,000 cells/mL whereas fat and SNF shall not be <3.35% and 8.25%, respectively [19].

### Milk sampling and milk quality data

A pooled BTM sample was collected monthly from each farm at milk collecting facilities, which was a common representative of its corresponding dairy farm. The milk sampling was performed by livestock officers. First, a BTM sample (8-10 mL) from each farm was collected into a sterile tube. Next, tubes were kept in cool containers with ice blocks and sent to a laboratory within 4 h. Then, milk samples were analyzed to determine milk composition, including fat, SNF, protein, and lactose, using the Fourier-transformed Infrared Spectroscopy (MilkoScan FT6000; Foss Electric, Hillerød, Denmark). Subsequently, the SCC were quantified by Fossomatic 5000 (Foss Electric) operated by the Regional Veterinary Research and Development Centers in the northern and the central regions [20].

In this study, we used milk quality datasets from Veterinary Research and Development Centers of DLD that included monthly data on milk composition and SCC levels of BTM samples taken from dairy farms in the provinces of Chiang Mai (n=18,541), Saraburi (n=11,065), and Lopburi (n=7471). The milk quality data were collected from 1580 farms in five dairy cooperatives in Chiang Mai and 1562 farms in three dairy cooperatives in Saraburi and Lopburi Provinces. Thus, the number of milk samples tested and the number of dairy farms in the two regions were fairly similar. Figure-1 depicts dairy cooperative geographical locations. Following the PPBMQ parameters, only fat, SNF, and SCC data were used. Notably, the SCC values were used as SCC*1000. For example, SCC=320*1000 cells/mL were defined as SCC=320 [21,22].

### Payment programs based on milk quality

According to the PPBMQ, milk is priced based on levels of quality and divided into the base, bonus, and deduction payment classes (Table-1). With the PPBMQ, the higher the milk quality sold, the higher the price paid to the farmers. For example, if SNF in raw BTM is between 8.35 and 8.49, the farmer receives the base price. If the SNF is 8.5-8.69, the farmer is paid 300 baht (approximately 9.23 USD) along with the base price for every 1000 kg of raw milk. However, if SNF is ≤8.25, the farmer is deducted 600 baht (approximately 18.45 USD) from the base price for every 1000 kg of raw milk sold.

**Table 1:** Payment class is made by addition, deduction, or neither through the payment program based on milk quality of bone marrow transplant in Thailand.

Component	Range	Class	Payment class: Addition (+)/deduction (–)/neither addition nor deduction (--)
Fat	≥4	A++	+400 (12.84)*
	3.99-3.8	A+	+300 (9.63)
	3.79-3.6	A	+200 (6.42)
	3.59-3.4	B	--
	3.39-3.2	C	–200 (6.42)
	≤3.2	D	–400 (12.84)
Solids not fat	≥8.7	A+	+600 (19.26)
	8.69-8.5	A	+300 (9.63)
	8.49-8.35	B	--
	8.34-8.25	C	–300 (9.63)
	≤8.25	D	–600 (19.26)
Somatic cell counts	≤200,00	A++	+500 (16.05)
	200,001-300,000	A+	+300 (9.63)
	300,001-400,000	A	+200 (6.42)
	400,001-500,000	B	--
	500,001-700,000	C	–200 (6.42)
	700,001-1,000,000	D	–300 (9.63)
	>1,000,000	E	–500 (16.05)

* Baht ($US) per 1000 kg

Due to different milk compositions from each BTM, the farmer may receive an additional price for one milk composition (e.g., fat) whereas receiving a deduction price for another composition (e.g., protein). Given that, the final price (FP) was the sum of all individual milk composition prices after addition or deduction, or none of these prices were applied to each component. Thus, if the FP is greater than zero (additional payment), a dairy farmer is placed into a gain-benefit group. If the FP is less than zero (deduction payment), a farmer is placed into a loss-benefit group. If the FP is equal to zero (base payment), a dairy farmer is placed into a no-benefit group. For example, given that dairy farmers received a deduction price from Class C of SCC (–200) but received an additional price from Class A^+^ of fat (+300) based on the PPBMQ shown in Table-1, and the dairy farmers finally gained the benefit and then they were classified into a gain-benefit group.

### Statistical analysis

#### Descriptive statistics

Based on the FP, BTM samples were first categorized into different payment classes including addition, deduction, and neither of those classes, and then the percentages of BTM samples belonging to these classes were calculated. Moreover, primary patterns based on the combination of the payment classes and the milk components in BTM for each region were identified. Several functions from R statistical software and its packages, including “dplyr” and “ggplot2” [23] were used to summarize, analyze, and visualize the data.

### Comparisons of proportions

To test the hypothesis of whether the proportion of dairy farmers receiving benefits from the PPBMQ for each month between those in the northern and central regions are different, a mixed-effects logistic regression model [24,25] was used. Since farms are located in different regions, geographical factors should be concerned. Thus, the cluster effects of farms nested in each region were included in the model. The analysis defined the region and month variables as fixed effects whereas an individual farm was defined as a random effect. The statistical model is expressed as:


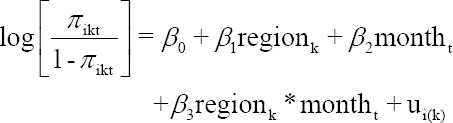


where *π_ikt_*=*p*(*y_ikt_*=1) denotes the probability of *i*^th^ dairy farmer clustered in *k*^th^ region (1=northern, 2=central region) receiving bonus price in *t*^th^ month (month=1, 2,…, 24), *region_k_* = fixed effect due to region, *time_t_*=fixed effect due to month that dairy farmer receives the payment, region_k_*time_t_= interaction effects between *region_k_* and *time_t_*, β_1_, β_2_, and β_3_=coefficients corresponding to fixed-effect variables, and *u_i(*k*)_*=random effect of farm *i*^th^ clustered in *k*^th^ region. It was assumed that *u_i(*k*)_*~*NID* (0, σ*_u_* ^2^).

For multiple comparisons, the differences in the proportion of dairy farmers gaining benefits between the central and northern regions were tested for each month using Tukey’s method. The mixed-effects logistic regression model analysis was performed using R with “lme4” package [26], and the multiple comparisons were done by functions from “emmeans” package [27]. The level of significance for statistical analysis was set as a=0.05.

## Results

### Descriptive statistics

**Figure-2:**
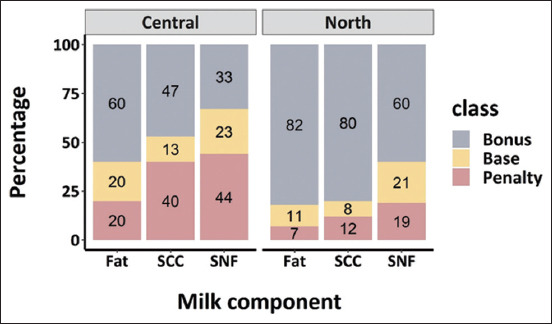
Percentage of bulk tank milk (BTM) in different payment classes for various milk components, including fat, somatic cell count, and solids not fat. BTM samples were collected from the central and northern region of Thailand. The same pricing system was used for both regions.

Percentages of BTM in each class by each milk component are presented in Figure-2. More than 80% of BTM in the northern region received bonus prices from fat or SCC components, whereas a lower percentage was found for those from the central region. For the central region, the percentages of BTM received a penalty price due to SCC and SNF were 40% and 44%, respectively.

**Figure-3:**
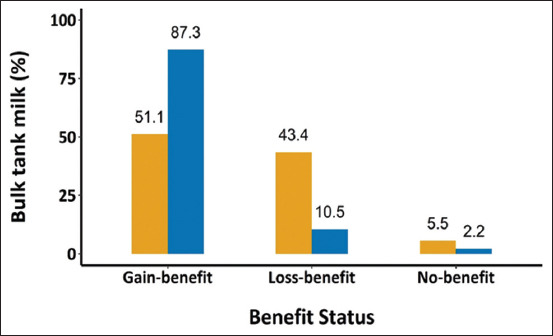
The percentage of bulk tank milk taken from the central (yellow) and northern (blue) regions categorized by the final payment groups, including gain-benefit, loss-benefit, and no benefit groups.

Overall, 73% of BTM samples were categorized into the additional payment class based on the FP. Figure-3 shows that nearly 40% of BTM samples from the central region and <10% of those from the northern region have belonged to the deduction payment class. However, more than 80% of BTM samples from the northern region were categorized into the additional payment class.

The top five patterns of milk components based on the payment program of dairy farmers in the northern and central regions are shown in Tables-2 and 3, respectively. The predominant pattern of milk components in both regions was categorized under bonus classes. However, the percentage of this pattern in the northern region (42%) was approximately 4 times higher than that in the central region (10.7%).

**Table 2:** Primary patterns of payment classes and milk components in bulk tank milk from the central region of Thailand.

Fat	Solids not fat	Somatic cell counts	Percentage
Bonus	Bonus	Bonus	10.7
Bonus	Bonus	Penalty	10.2
Bonus	Penalty	Penalty	9.9
Bonus	Penalty	Bonus	9.1
Penalty	Penalty	Bonus	7.2

Green and red colors indicate bonus and penalty group, respectively

**Table 3:** Primary patterns of payment classes and milk components in bulk tank milk from the northern region of Thailand.

Fat	Solids not fat	Somatic cell counts	Percentage
Bonus	Bonus	Bonus	42
Bonus	Base	Bonus	13.6
Bonus	Penalty	Bonus	9.3
Bonus	Bonus	Penalty	6.11
Base	Bonus	Bonus	4.1

Green, yellow, and red colors indicate bonus, base, and penalty group, respectively

When considering other patterns, the top three northern samples included bonuses from both fat and SCC. In contrast, two of the top three most common patterns in central region samples contained the penalty due to the SCC. A penalty due to SCC was observed in the second and the third top-three patterns of BTMs from the central region, indicating the SCC was involved in a price reduction for several dairy farmers in this region.

### Proportions of dairy farmers gaining benefits

**Figure-4:**
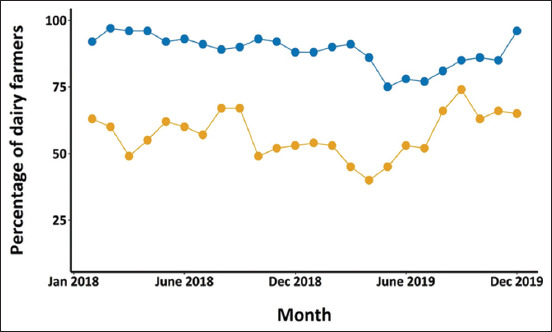
Monthly percentage of dairy farmers in the central (yellow dots) and northern (blue dots) regions gaining benefit from the milk payment system.

Throughout the study period, the proportion of dairy farmers in the northern region earning benefits due to the PPBMQ was statistically higher than those in the central region, except for September 2019 (Figure-4). In a 24-month study, the proportion of dairy farmers in the northern region was remarkably gaining benefits of more than 80% for 21 months. In contrast, those in the central region were gaining <75% for all months. Furthermore, the proportion of dairy farmers in the northern region receiving the benefit appears to be less variable than those in the central region.

## Discussion

PPBMQs are often based on a bonus, a penalty, or both [15], with the latter being utilized in Thailand. The main approach of the PPBMQ is to increase the financial incentive for dairy farmers who produce high-quality milk. Nevertheless, the results showed that a substantial proportion of dairy farmers lost benefits due to the PPBMQ, especially for dairy farmers in the central region. Throughout the study period, the central region had a higher proportion of dairy farmers who lost benefits when compared with those in the northern region. This finding provided evidence that dairy farmers in the central region face a substantial challenge in producing high-quality milk to attain a benefit price. SCC was the most important milk component contributing to the penalty for dairy farmers in the central region. Thus, the implementation of a rigorous mastitis control program should be a primary priority for dairy farmers in the central region because the high levels of bulk milk somatic cell count are associated with mastitis problems in dairy herds [28-31]. Furthermore, although most dairy farmers in both regions could produce milk with high fat and earn the bonus, many dairy farmers in both regions had some challenges producing milk with SNF that fall into the bonus class. Because the SNF is composed of protein, lactose, minerals, vitamins, and a few other components presented in milk, increasing these components results in an increase in the SNF. The levels of lactose, vitamins, and minerals in milk are constant and not subject to more changes in nutritional value by manipulation [32]. Thus, the appropriate strategy to increase SNF levels in milk is to feed dairy cows with sufficient quantities of dietary protein from a balanced diet of degradable and undegradable protein [32,33]. Moreover, in the long-term strategy, improving the genetic qualities of dairy cows in the herd will increase the farm’s milk quality as genetic structure of dairy cows affects milk yield and milk quality [34,35].

Mostly, dairy farmers are eager to improve milk quality to gain a higher price [36]. Nevertheless, improving the milk quality usually requires an additional cost. For instance, to enhance milk compositions such as protein and fat are necessary to provide a high-quality cow feed along with proper nutritional management on the farm [37], which incurs an extra cost. In addition, to reduce the SCC levels, rigorous mastitis control programs must be undertaken, which include maintaining or replacing milk equipment, utilizing proper udder disinfectants, and routinely checking a herd’s mastitis status [38-40]. These programs often demand additional budgets. Given that, some farmers may not decide to improve their milk quality if the cost of improving quality exceeds the benefit to them. Therefore, some dairy farmers may receive a price that is lower than the base price on a regular basis. This situation may not be harmful if such farmers still earn a profit from farming. Unfortunately, some dairy farmers may lose their total profits several times each year and be unable to keep their farming business. To mitigate this problem, we suggested that authorities and stakeholders collaborate to assist such dairy farmers in improving or enhancing milk quality while maintaining profitability through the provision of technical, financial, and other support.

The PPBMQ is reported as an important factor in improving the profitability and sustainability of dairy farmers [15,16]. For example, India’s largest milk cooperative, the Kaira District Co-operative Milk Producers’ Union (Amul), bases its payment program on the fat and milk yield provided to the dairy. Rather than increasing each individual member’s ability to produce more milk, the cooperative’s goal is to grow the number of milk producers (e.g., by getting a better breed). As a result, the farmer receives a bonus from the cooperative at the end of the year, which is based on the value of the milk the farmer has contributed to the cooperative [41]. A study in Czech demonstrates the impact of various pricing systems on the economic values of milk traits (e.g., fat, protein, and SCC). In such a study, marginal economic values were determined and the most suitable payment system for Czech conditions is purposed [42]. As discussed earlier, the PPBMQs may be bonuses or penalties, or both payment systems. Some studies indicated that the PPBMQ based on penalties is the most effective in motivating dairy farmers to improve their BTM qualities [43,44]. A study in Brazil suggests using a penalization system exclusively used in the case of dairy farmers who do not realize the importance of the payment in terms of money lost per milk liter [14]. Nevertheless, it was suggested that a proper payment system should be straightforward for dairy farmers to adopt. Such a system can motivate the farmer to improve their BTM to increase profits [45]. For Thailand, the assessment of the existing PPBMQ and its impact on farmer economics and the interaction of dairy farmers with the PPBMQ have not been explored yet; thus, these topics should be investigated for follow-up studies.

Benefits received by dairy farmers determined in this study do not indicate the farmers’ profit. Some dairy farmers who lose benefits from the PPBMQ may still earn from their dairy farming operations.

## Conclusion

The study was the first to quantify the proportions of dairy farmers for different payment groups, including benefit, loss-benefit, and no benefit, based on Thailand’s PPBMQ. Our results highlighted that the proportion of dairy farmers in the central region receiving the benefit from the PPBMQ was lower than those in the northern region for the entire study period. The high level of SCC in BTM was the main variable resulting in a penalty for dairy farms in the central region. We suggest that authorities and stakeholders in the dairy sector should enhance their support for such dairy farmers to improve milk quality and increasing their benefits. Notably, because the data used in this study came from only two regions, future studies should include data from all dairy farming regions throughout the country to accurately represent the overall effects of PPBMQ on all dairy farmers in Thailand.

## Data Availability Statement

Supplementary data can be available from the corresponding author on a reasonable request.

## Authors’ Contributions

CS: Methodology, formal analysis, data curation, writing - review and editing, and visualization. KJ: Conceptualization, methodology, validation, formal analysis, visualization, supervision, and investigation. KK: Software, formal analysis, and visualization. NK: Methodology, validation, resources, investigation, data curation, and writing - review and editing. MM: Investigation, resources, and visualization. OA: Investigation and resources. VP: Conceptualization, methodology, software, validation, formal analysis, investigation, resources, data curation, writing - original draft preparation, writing - review and editing, visualization, supervision, project administration, and funding acquisition. All authors read and approved the final manuscript.
